# Emergency department visits among First Nations female adults in Alberta: a population-based study

**DOI:** 10.1007/s43678-025-00927-0

**Published:** 2025-08-21

**Authors:** Elenna LaPlante, Rhonda J. Rosychuk, Kimberley D. Curtin, Cheryl Barnabe, Katherine Rittenbach, Brian R. Holroyd, Lea Bill, Bonnie Healy, Eunice Louis, Patrick McLane

**Affiliations:** 1 University of Alberta, Edmonton, AB Canada; 2 University of Calgary, Calgary, AB Canada; 3 Alberta First Nations Information Governance Centre, Calgary, AB Canada; 4 Alberta Health Services (formerly), Edmonton, AB Canada; 5 Blackfoot Confederacy (formerly), Standoff, AB Canada; 6 Maskwacis Health Services, Maskwacis, AB Canada

**Keywords:** First Nations, Health Services, Premières nations, Services de santé

## Abstract

**Objective:**

Emergency departments (EDs) serve as a critical first access point for receiving healthcare for many First Nations members. However, ED experiences differ between First Nations and non-First Nations patients. Our objective is to quantify differences in ED visit characteristics for First Nations female and non-First Nations female adult patients in Alberta.

**Methods:**

We used healthcare administrative data from April 1, 2012 until March 31, 2017 linked to First Nations identifying data. We included all female patients aged 18–54 with ED encounters in Alberta, Canada. We extracted patient characteristics (e.g., age, gender, First Nations status) and ED visit characteristics (e.g., day of week, acuity, diagnosis, disposition). Descriptive statistics were calculated for each of the population groups (i.e., First Nations female and non-First Nations female patients). Mixed effects modeling statistical analyses were conducted to account for clustering when assessing for significant differences between population groups.

**Results:**

First Nations female patients who had ≥ 1 ED visit used the ED more compared to non-First Nations female patients (median 5, [IQR 2, 10]) vs. (median 2, [IQR 1, 4]), and a higher proportion of First Nations female visits resulted in admission (6.0% vs. 4.9%, *p* < 0.0001). First Nations female patients had a higher proportion of their visits diagnosed as related to unspecific findings, infection, cancer, obstetrical conditions, substance misuse/addictions, and mental health.

**Conclusion:**

Findings suggest a lack of access to culturally safe primary and specialty care that would allow treatment in community and support discharge from acute care. ED providers must understand the conditions that underly First Nations women’s visits to take a culturally safe approach that does not blame patients for the number or type of ED visits they require.

**Supplementary Information:**

The online version contains supplementary material available at 10.1007/s43678-025-00927-0.

## Résumé

**Objectif:**

Les services d’urgence (SU) constituent un premier point d’accès essentiel aux soins de santé pour les membres des Premières Nations. Cependant leurs expériences different en comparison aux patients de la population générale. Notre objectif est de quantifier les différences dans les caractéristiques des visites aux salles d’urgences entre les patientes adultes des Premières Nations et celles des autres populations en Alberta.

**Méthodes:**

Nous avons utilisé les données administratives sur les soins de santé du 1er avril 2012 au 31 mars 2017 coupllées aux données d’identification des Premières Nations. Nous avons inclus toutes les patientes âgées de 18 à 54 ans qui ont eu des rencontres avec une salle d'urgence en Alberta, au Canada. Nous avons extrait les caractéristiques des patients (p. ex., âge, sexe, statut des Premières Nations) et les caractéristiques des visites aux salles d’urgence (p. ex., jour de la semaine, acuité, diagnostic, disposition). Des statistiques descriptives ont été calculées pour chaque groupe de population (c’est-à-dire femmes des Premières Nations et les patientes non membres). Des analyses statistiques par modélisation à effets mixtes ont été réalisées afin de tenir compte du regroupement lors de l’évaluation des différences significatives entre les groupes de population.

**Résultats:**

Les patientes des Premières Nations ayant eu ≥ 1 visite à une salle d’urgence avaient plus de visites compaé à ceux non- Premières Nations (médiane de 5 visites par individuelle, [IQR 2, 10] contre médiane de 2, [IQR 1, 4]), et et ont plus souvent exiger l’admission (6,0 % contre 4,9 %, p<0,0001). Les patientes des Premières Nations ont été diagnostiquées plus souvent pour des observations non spécifiques, les infections, le cancer, des conditions obstétricales, la toxicomanie et des problems de santé mentale.

**Conclusion:**

Les résultats indiquent un manque d’accès à des soins primaires et spécialisés culturellement sécuritaires qui permettraient le traitement dans le milieu communautaire et qui faciliteraient la sortie des soins de courte durée. Les professionelles des soins de santé d’urgence doivent comprendre les conditions qui sous-tendent les visites des femmes des Premières Nations afin d’adopter une approche culturellement sécuritaire qui ne blâme pas les patients pour le nombre ou le type de visites d’urgence dont ils ont besoin.

## Clinician’s capsule

**Table:** 

***What is known about the topic?***
Although EDs are a healthcare access point for First Nations, there are differences between care provided to First Nations and non-First Nations patients.
***What did this study ask?***
This study quantified differences in ED visit characteristics for First Nations female and non-First Nations female adult patients in Alberta.
***What did this study find?***
Female First Nations patients use the ED more per patient, had a higher proportion of admissions, and different distribution of diagnoses.
***Why does this study matter to clinicians?***
Access to culturally safe primary and specialty care could address high ED use among First Nations women.

## Introduction

Colonialism inflicted upon Indigenous peoples in Canada has led to substantial health disparities between Indigenous and non-Indigenous people [1–3]. Fragmented healthcare services between provincial and federal governments result in limited access to healthcare systems for First Nations peoples [1, 4]. Higher emergency department (ED) use by Indigenous peoples could reflect a lack of primary care options [5–7].

The term First Nations denotes one of three Indigenous groups, alongside Métis and Inuit, holding Aboriginal rights in Canada [8]. Evidence in Alberta suggests that ED experiences differ between First Nations and non-First Nations patients, as demonstrated by higher rates of leaving without completing care [5], and lower odds of being assigned high acuity triage scores across multiple diagnoses [9, 10]. These race-based differences may be compounded by gender-based differences [4]. Colonial and patriarchal systems create conditions where First Nations women are subject to “discrimination within discrimination” based on their race and gender [11]. These conditions influence healthcare encounters [12]. As a political-social system, patriarchy perpetuates colonial ideals and limits women’s agency and authority [13].

There is a lack of data describing ED utilization for First Nations women [5]. Because First Nations women are subject to racialized and gendered inequities [14, 15], First Nation- and gender-specific analysis is required to identify gaps in care and potential solutions. Our objective is to quantify differences in ED visit characteristics for adult First Nations and non-First Nations female patients in Alberta.

## Methods

### Study design & time period

This descriptive analysis is a part of a larger project [5, 9, 10], co-developed with First Nations partners, including the Alberta First Nations Information Governance Centre (AFNIGC), University researchers, and health system employees. The AFNIGC protects the principles of OCAP® (Ownership, Control, Access and Possession of data), and First Nations right of self-determination in research [16]. An Elder Advisory group guided the project, and First Nations team members participated in all aspects of the study design and interpretation of findings. Research agreements between partners govern dissemination and publication of results.

The study design is a population-based cohort including ED visits in Alberta from April 1, 2012 until March 31, 2017 [5]. The University of Alberta Health Research Ethics Board (Pro 00082440) granted ethics approval.

### Study setting and population

We analyzed data from visits to 111 facilities, including 95 EDs, seven urgent care centres, and nine ambulatory care centers where unscheduled medical care is provided to patients [5].

The identification of First Nations persons in the Alberta Health Care Insurance Plan (AHCIP) Population Registry is possible because healthcare premium payments were paid by the federal government for registered First Nations persons until 2009 [17]. Alberta Health has maintained this dataset by removing deceased persons, and by adding individuals (e.g., children) who have an AHCIP account where the main registrant was identified as First Nations [17].

This analysis focused on females, aged 18–54 years at the time of ED visit. The age range was determined by First Nations partners. Due to the lack of gender identifiers collected by the *National Ambulatory Care Reporting System (NACRS)*, reported biologic sex is utilized as a proxy for gender.

### Data sources & variables

The NACRS reports data related to patient demographics and emergency care visits [18] including patient age, biologic sex, time/day of visit, arrival mode (i.e., air ambulance), Canadian Triage Acuity Scale (CTAS) [19], and disposition. CTAS rates the acuity of the chief complaint and enables treatment prioritization of the most severe cases [19]. De-identified quantitative administrative data from Alberta Health Services (AHS) and the Ministry of Health were obtained [20]. Data custodians completed person-level linkages between datasets. AHS provided variables for the Charlson Comorbidity Index plus hypertension, primary codes for Episode Disease Category, travel time and distance. The Charlson Comorbidity Index assesses patient comorbidities based on adjusted mortality and resource use [21]. Visit diagnoses were categorized using the 3M Episode Disease Category system [22], and further grouping was completed by clinician team members (as described in [5]).

### Data analysis

Numerical summaries, including means, medians, standard deviations, IQRs [represented as 25th percentile, 75th percentile], and counts (with percentages) report the patient demographics and emergency visit data by First Nations status and gender. Mixed effects logistic regression models determined the significance of the association between each variable separately and First Nations status by using a random effect for patient to account for the dependency of multiple visits by individual patients. Statistical analyses were conducted in R [23] and SAS [24]. A *p*-value < 0.05 was considered statistically significant.

## Results

Our dataset included 41,986 (5.3%) unique female First Nations patients and 748,935 (94.7%) unique non-First Nations female patients. Table 1 describes visit characteristics for First Nations and non-First Nations females. Female First Nations patients had 366,184 (11.5%) of the 3,177,079 female adult ED visits during the study period. First Nations females that use the ED use it more (median 5, [IQR 2, 10]) compared to non-First Nations female ED patients (median 2, [IQR 1, 4]) over the study period (see also Appendix A).

**Table 1:** Emergency care visit characteristics for First Nations females and non-First Nations females aged 18–54 years^a,b^

Variable	First Nations female (*n* = 366,184)	Non-First Nations female (*n* = 2,810,895)
ED visits per patient (over study period)		
Median, IQR	5 [2.0, 10.0]	2 [1.0, 4.0]
Age (years)^c^		
Mean, SD	33.8 (10.4)	34.4 (10.5)
Travel time (minutes)^d^		
Mean, SD	17.1 (25.2)	9.7 (9.2)
Travel distance (km)^d^		
Mean, SD	18.1 (32.1)	7.3 (10.6)
Visit day (%)		
Monday-Friday	72.2	72.1
Saturday-Sunday	27.8	27.9
Visit time (%)		
8:01–16:00	44.1	49.1
16:01–00:00	43.2	39.0
00:01–8:00	12.7	11.9
Ambulance arrival (%)		
Arrive by ambulance—Air	0.1	0.0
Arrive by ambulance—Ground	15.6	6.1
Arrive by ambulance—Combined	0.3	0.0
Not admitted by ambulance	84.0	93.8
Triage level (%)		
CTAS 1—resuscitation	0.4	0.2
CTAS 2—emergent	7.8	10.5
CTAS 3—urgent	29.9	36.0
CTAS 4—less urgent	39.5	38.1
CTAS 5—non-urgent	17.7	12.0
No CTAS assigned	4.8	3.2
Charlson Comorbidity Index Score (%)		
0	68.8	81.8
1	20.5	12.9
2	5.6	3.0
3	2.2	1.1
4	1.0	0.4
5	0.4	0.2
6	0.5	0.1
7 or higher	1.3	0.6
Top 10 episode disease category (%) (by FN count)		
Infection	15.6	14.5
Trauma and injury	12.9	14.2
Unspecific signs, symptoms and findings	10.7	9.2
Gastrointestinal conditions	11.1	13.2
Breast, obstetrics, and gynecology	9.7	10.1
Cancer	4.6	4.0
Substance misuse/addictions	3.9	0.9
Mental health	3.8	3.1
Respiratory conditions	3.4	3.3
Musculoskeletal and arthritis conditions	3.2	3.4
Disposition (%)		
Discharged	84.7	89.1
Admitted	6.0	4.9
Transferred	1.9	1.5
Left without being seen	4.9	3.3
Left against medical advice	2.5	1.1
Deceased	0.0	0.0

^a^All difference between FN female and non-FN female are significant at the *p* < 0.05 level except for visit day (*p* = 0.12)

^b^ < 30 additional visits by FN and non-FN female patients had “Other” recorded as the biologic sex of the patient, these patients were included in the female counts

^c^Analysis restricted to ages 18–54 years

^d^Travel time and travel distance had missing data, for these two variables for First Nations and non-First Nations females *n* = 349,706 and *n* = 2,709,703, respectively

First Nations females visits were more commonly (57.2%) triaged as CTAS 4 (less urgent) and CTAS 5 (non-urgent), compared to 50.1% for non-First Nations. First Nations females were more likely to present in the evening/night hours (55.9% vs. 50.9%). First Nations female visits were assocated with more comorbitities. The top 10 Episode Disease Category by First Nations count comprised 78.7% of all First Nations visits. Relative to non-First Nations females First Nations females had a higher proportion of their visits diagnosed as related to infections (15.6% vs. 14.5%), unspecific signs, symptoms and findings (10.7% vs. 9.2%), cancer (4.6% vs. 4.0%), substance misuse/addictions (3.9% vs. 0.9%), and mental health concerns (3.8% vs. 3.1%). Trauma and injury; gastrointestinal conditions; breast, obstetrics and gynecological conditions; and musculoskeletal and arthritis conditions made up a higher percent of non-First Nations than First Nations visits. Obstetrical conditions were investigated as an area of importance to the Elder Advisory. Within this category of visits, a higher proportion of First Nations female visits were for an obstetrical diagnosis (6.3% vs. 5.7%, *p* < 0.0001). In addition, a higher proportion of First Nations female visits resulted in the patient leaving without being seen (4.9% vs. 3.3%,), leaving against medical advice (2.5% vs. 1.1%), admission (6.0% vs. 4.9%), and transfer (1.9% vs. 1.5%).

Table 2 details obstetrical diagnosis for First Nations and non-First Nations females. Among obstetrical presentations, First Nations females had a higher proportion of visits for supervision of pregnancy and false labor while non-First Nations patients had a higher proportion of complications including hemorrhage, hyperemesis gravidarum, and threatened abortion.

**Table 2:** Top 10 specific obstetrical diagnosis for First Nations female and non-First Nations female patients aged 18–54 years as percentages of all obstetric diagnoses for each population group^a^

Description of obstetrical diagnoses	First Nations female *n* = 23, 204	Non-First Nations female *n* = 160,324
Supervision of other normal pregnancy; supervision of normal pregnancy, unspecified; supervision of normal first pregnancy; or pregnancy confirmed	25.6%	16.6%
Threatened abortion, antepartum condition or complication	9.4%	13.6%
Hemorrhage in early pregnancy, unspecified, antepartum condition or complication	8.3%	14.1%
Other specified diseases and conditions complicating pregnancy, childbirth and the puerperium, antepartum condition or complication	7.1%	7.1%
Mild hyperemesis gravidarum, antepartum condition or complication	4.2%	7.1%
False labor at or after 37 completed weeks of gestation, antepartum condition or complication	3.5%	0.7%
False labor before 37 completed weeks of gestation, antepartum condition or complication	2.1%	0.6%

^a^All difference between FN female and non-FN female are significant at the *p* < 0.05 level

## Discussion

### Interpretation of findings

This study compares the frequency and characteristics of ED visits for adult female First Nations and non-First Nations patients in Alberta, Canada. First Nations patients had more ED visits per person, but had less acute triage scores and more admissions and transfers than non-First Nations patients. More visits by First Nations adult females also resulted in leaving without completing care. Differences in many ED visit characteristics and diagnoses likely stem from inequitable access to healthcare and systemic or overt racism rather than First Nations women’s choices about where and how to access care. Lack of access to culturally safe primary and specialty care would contribute to higher ED use. Pregnant First Nations patients had a higher proportion of ED visits related to false labor and supervision of pregnancy compared to pregnant non-First Nations patients. Lack of consistent access to culturally safe prenatal care may result in an increased use of the emergency department for obstetrical presentations [25]. Lower triage scores may reflect presentations for conditions that might otherwise be cared for in outpatient settings. However, the combination of lower CTAS scores and higher admission rates may be evidence of ongoing discrimination and under-triage of First Nations patients. Indigenous patients are frequently inaccurately stereotyped as misusers or overusers of acute care [26–29]. Higher admissions among female First Nations patients may also relate to lack of services supporting discharge, advanced presentations of conditions that were untreated or under-treated in outpatient settings, or advanced presentations related to delayed care because of initial ED avoidance. Leaving without completing care may be a result of discrimination and racism within the ED setting [9]. While our data show higher use of ED among First Nations female adult patients, and higher use for certain diagnoses, this should be understood as a result of systemic conditions rather than choices individuals make about when and where to access care.

### Previous studies

Our findings align with previous literature. Access to quality healthcare services is a substantial problem for First Nations women. Broader literature implies a link between limited access to quality primary care and higher ED use [5–7, 30, 31]. The Canadian healthcare system provides inadequate and inconsistent healthcare to Indigenous peoples [32, 33]. Indigenous peoples often have to travel to receive care, removing them from their community, and disrupting management of their health [32, 33]. Presentation in the evening/overnight as found in our study may relate to transport issues, and was previously theorized by Elders to be related to availability of vehicles/drivers [5].

First Nations women may also avoid the health system due to negative experiences. For example, forced sterilization [15], experiences with child protection services [34], and negative stereotyping all affect the decision to seek healthcare [1]. Evidence of racism and discrimination have been documented in Alberta EDs [9, 35]. In a recent survey, explicit anti-Indigenous prejudice was reported by Albertan physicians [36]. Negative care encounters may result in delays in seeking care, and greater probability of ED use when condtions become intolerable or severe.

### Strengths and limitations

A key strength of this research is co-interpretation of the data with First Nations partners. Partners could interpret our findings through their own experience and knowledge of their communities, adding validity to interpretations.

As our data sources did not systematically capture gender, we used biologic sex as a proxy for gender. Although this is a significant limitation, a large degree of overlap between female sex identity and identifying as a woman is expected. The 2021 census reported approximately 99.5% of First Nations people in Canada identify as cis-gender [37]. We did not undertake separate analyses of presentations in urban and rural hospitals. Prior literature suggests that rural hospitals have a different case mix and distribution of CTAS scores compared to urban hospitals, and differential attendance at rural or urban hospitals between First Nations and non-First Nations patients may confound the observed association between First Nations status and these study outcomes. However, our previous work [10] did not identify an interaction between First Nations status, triage scores and distance traveled to hospital. A further limitation is that non-status First Nations persons, and status First Nations persons who arrived to Alberta after 2009, are counted as non-First Nations persons in the data. This misclassification may lead to underestimation of systematic differences in care provided to First Nations and non-First Nations patients. In addition, Métis and Inuit persons are recorded as non-First Nations in the data, so we could not quantify differences in care provided to these Indigenous groups [38].

### Health systems/clinical implications

Increasing access to services on-reserve or in community is important for ensuring a connection to family and cultural supports [32, 33], and could reduce ED visits and admissions. In addition, ED providers must understand the factors that drive ED use for First Nations women, and avoid stereotyping or blaming individual patients for the number or type of ED visits they require. It is important to highlight the necessity of cultural safety in emergency care delivery. Partnering with local Indigenous communities to optimize culturally safe initiatives in the ED is fundamental to the relevance and effectiveness of these initiatives [39].

### Research implications

Research on women’s health should not simply document, but contribute to strategies that promote equity [12]. The experiences of First Nations women in the emergency system is complex and affected by access to the broader healthcare system. Further research is needed on First Nations women’s access to, experiences in, and outcomes of primary and specialty care in relation to ED use. Understanding is also needed on ED care during the whole life course, as well as the experiences of gender-diverse individuals in the ED. In addition, research using more recent data to better reflect the current conditions of ED care, including the post-pandemic care environment, would be of value.

## Conclusion

Female First Nations patients use the ED more frequentlycompared to non-First Nations female patients. First Nations patients are admitted or transferred more frequently, and leave the ED without completing care more frequently, compared to non-First Nations patients. ED use and admissions may be explained by a lack of accessible, culturally safe primary and specialty care that would allow treatment in community and support discharge from acute care. First Nations women leaving ED without completing care may be due to negative experiences within EDs.

## Supplementary Information

Below is the link to the electronic supplementary material.

Supplementary material 1 (DOC 85.5 kb)

## Data Availability

The original data sources for this study are owned by Alberta Health Services and Alberta Health. Readers may contact the corresponding author for more information.
