# Supplementary material for: Emergency department visits among First Nations female adults in Alberta: a population-based study
Source: CJEM. 2025 Aug 21;27(9):716–23. doi: 10.1007/s43678-025-00927-0 (PMC12484333; doi:10.1007/s43678-025-00927-0)
Supplement: Supplementary file 1 — Supplementary material 1 (DOC 85.5 kb) [file 43678_2025_927_MOESM1_ESM.doc]

**Emergency department visits among First Nations female adults in Alberta: A population-based study**

**APPENDIX 1: Number of Visits per Patient per Fiscal Year for First Nations Females and non-First Nations Females, aged 18-54 years.**

| **Number of Visits** | **2012/13** | | **2013/14** | | **2014/15** | | **2015/16** | | **2016/17** | |
| --- | --- | --- | --- | --- | --- | --- | --- | --- | --- | --- |
|  | **First Nations Females**  **(%)** | **Non-First Nations Females (%)** | **First Nations Females (%)** | **Non-First Nations Females (%)** | **First Nations Females (%)** | **Non-First Nations Females (%)** | **First Nations Females (%)** | **Non-First Nations Females (%)** | **First Nations Females (%)** | **Non-First Nations Females (%)** |
| 1 | 39.5 | 57.9 | 40.0 | 58.2 | 40.6 | 58.8 | 39.9 | 59.4 | 41.1 | 60.1 |
| 2 | 21.1 | 21.3 | 21.3 | 21.2 | 20.6 | 21.0 | 21.5 | 20.8 | 21.2 | 20.6 |
| 3 | 12.7 | 9.2 | 12.4 | 9.1 | 12.5 | 9.0 | 12.5 | 8.8 | 11.8 | 8.6 |
| 4+ | 26.6 | 11.7 | 26.3 | 11.5 | 26.3 | 11.2 | 26.1 | 11.0 | 26.0 | 10.6 |
